# Development of herbicide-resistant peanut via prime editing and optimization of editing efficiency

**DOI:** 10.1093/plphys/kiag116

**Published:** 2026-03-25

**Authors:** Lulu Xue, Pengyu Qu, Huanhuan Zhao, Han Liu, Xiaona Li, Xiaobo Wang, Bingyan Huang, Suoyi Han, Xiaodong Dai, Wenzhao Dong, Lei Shi, Xinyou Zhang

**Affiliations:** Institute of Crop Molecular Breeding, Henan Academy of Agricultural Sciences/Key Laboratory of Oil Crops in Huang-Huai-Hai Plains, Ministry of Agriculture/Henan Provincial Key Laboratory for Oil Crops Improvement/National and Provincial Joint Engineering Laboratory for Peanut Genetic Improvement, Zhengzhou, Henan 450002, China; College of Agronomy, Shenyang Agricultural University, Shenyang, Liaoning 110866, China; Institute of Crop Molecular Breeding, Henan Academy of Agricultural Sciences/Key Laboratory of Oil Crops in Huang-Huai-Hai Plains, Ministry of Agriculture/Henan Provincial Key Laboratory for Oil Crops Improvement/National and Provincial Joint Engineering Laboratory for Peanut Genetic Improvement, Zhengzhou, Henan 450002, China; Institute of Crop Molecular Breeding, Henan Academy of Agricultural Sciences/Key Laboratory of Oil Crops in Huang-Huai-Hai Plains, Ministry of Agriculture/Henan Provincial Key Laboratory for Oil Crops Improvement/National and Provincial Joint Engineering Laboratory for Peanut Genetic Improvement, Zhengzhou, Henan 450002, China; Institute of Crop Molecular Breeding, Henan Academy of Agricultural Sciences/Key Laboratory of Oil Crops in Huang-Huai-Hai Plains, Ministry of Agriculture/Henan Provincial Key Laboratory for Oil Crops Improvement/National and Provincial Joint Engineering Laboratory for Peanut Genetic Improvement, Zhengzhou, Henan 450002, China; Institute of Crop Molecular Breeding, Henan Academy of Agricultural Sciences/Key Laboratory of Oil Crops in Huang-Huai-Hai Plains, Ministry of Agriculture/Henan Provincial Key Laboratory for Oil Crops Improvement/National and Provincial Joint Engineering Laboratory for Peanut Genetic Improvement, Zhengzhou, Henan 450002, China; Institute of Crop Molecular Breeding, Henan Academy of Agricultural Sciences/Key Laboratory of Oil Crops in Huang-Huai-Hai Plains, Ministry of Agriculture/Henan Provincial Key Laboratory for Oil Crops Improvement/National and Provincial Joint Engineering Laboratory for Peanut Genetic Improvement, Zhengzhou, Henan 450002, China; Henan Biological Breeding Center Co., Ltd., Zhengzhou, Henan 450002, China; The Shennong Laboratory, Zhengzhou, Henan 450002, China; Institute of Crop Molecular Breeding, Henan Academy of Agricultural Sciences/Key Laboratory of Oil Crops in Huang-Huai-Hai Plains, Ministry of Agriculture/Henan Provincial Key Laboratory for Oil Crops Improvement/National and Provincial Joint Engineering Laboratory for Peanut Genetic Improvement, Zhengzhou, Henan 450002, China; Henan Biological Breeding Center Co., Ltd., Zhengzhou, Henan 450002, China; Institute of Crop Molecular Breeding, Henan Academy of Agricultural Sciences/Key Laboratory of Oil Crops in Huang-Huai-Hai Plains, Ministry of Agriculture/Henan Provincial Key Laboratory for Oil Crops Improvement/National and Provincial Joint Engineering Laboratory for Peanut Genetic Improvement, Zhengzhou, Henan 450002, China; Henan Biological Breeding Center Co., Ltd., Zhengzhou, Henan 450002, China; Institute of Crop Molecular Breeding, Henan Academy of Agricultural Sciences/Key Laboratory of Oil Crops in Huang-Huai-Hai Plains, Ministry of Agriculture/Henan Provincial Key Laboratory for Oil Crops Improvement/National and Provincial Joint Engineering Laboratory for Peanut Genetic Improvement, Zhengzhou, Henan 450002, China; Henan Biological Breeding Center Co., Ltd., Zhengzhou, Henan 450002, China; Institute of Crop Molecular Breeding, Henan Academy of Agricultural Sciences/Key Laboratory of Oil Crops in Huang-Huai-Hai Plains, Ministry of Agriculture/Henan Provincial Key Laboratory for Oil Crops Improvement/National and Provincial Joint Engineering Laboratory for Peanut Genetic Improvement, Zhengzhou, Henan 450002, China; Henan Biological Breeding Center Co., Ltd., Zhengzhou, Henan 450002, China; Institute of Crop Molecular Breeding, Henan Academy of Agricultural Sciences/Key Laboratory of Oil Crops in Huang-Huai-Hai Plains, Ministry of Agriculture/Henan Provincial Key Laboratory for Oil Crops Improvement/National and Provincial Joint Engineering Laboratory for Peanut Genetic Improvement, Zhengzhou, Henan 450002, China; Henan Biological Breeding Center Co., Ltd., Zhengzhou, Henan 450002, China; The Shennong Laboratory, Zhengzhou, Henan 450002, China; Institute of Crop Molecular Breeding, Henan Academy of Agricultural Sciences/Key Laboratory of Oil Crops in Huang-Huai-Hai Plains, Ministry of Agriculture/Henan Provincial Key Laboratory for Oil Crops Improvement/National and Provincial Joint Engineering Laboratory for Peanut Genetic Improvement, Zhengzhou, Henan 450002, China; College of Agronomy, Shenyang Agricultural University, Shenyang, Liaoning 110866, China; Henan Biological Breeding Center Co., Ltd., Zhengzhou, Henan 450002, China; The Shennong Laboratory, Zhengzhou, Henan 450002, China

## Abstract

Prime editing of acetolactate synthase to introduce the W574L mutation confers broad-spectrum herbicide resistance in peanut, though efficiency remains suboptimal and requires further refinement.

Dear Editor,

Prime editor (PE) consists of an H840A Cas9 nickase (nCas9)-reverse transcriptase (RT) fusion protein and a prime editing guide RNA (pegRNA), which both specifies the target site and carries the desired edits. This system enables the introduction of all 12 types of point mutations as well as small insertions and deletions (indels), without requiring double-strand DNA breaks or donor DNA templates (Anzalone et al. 2019). These features make PE a powerful tool for plant research and crop improvement. It has been successfully applied in rice, maize, wheat and tomato (Jiang et al. 2020; Ni et al. 2023; Gupta et al. 2024; Vu et al. 2024), but not yet in the allotetraploid peanut (*Arachis hypogaea* L.). Since CRISPR-Cas systems and base editors are unable to target certain agronomically important mutations (Tuncel et al. 2025), PE represents an essential complementary genome editing platform for peanut. Acetolactate synthase (ALS) is a key enzyme in the biosynthesis of branched-chain amino acids (valine, leucine, and isoleucine) and serves as the target for many commercial herbicides (Duggleby et al. 2008). Mutations in ALS can confer herbicide resistance in various plant species (Han and Kim 2019).

To evaluate whether PE can introduce precise edits at endogenous genetic loci in peanut, we utilized a PE system that comprised a CaMV35S promoter-driven, plant codon-optimized nCas9 (H840A)-engineered Moloney murine leukemia virus (M-MLV) RT (D200N, L603W, T330P, T306K, W313F) fusion protein (Anzalone et al. 2019). A pegRNA was designed to introduce a W574L mutation (TGG to TTG) at the *A. hypogaea ALS2* (*AhALS2*) locus, a known herbicide-resistance mutation in plants (Han and Kim 2019). The pegRNA comprises a single guide RNA, a 13-bp RT template, and an 11-bp primer binding site sequence (Fig. 1a; Text S1; mutation site numbered based on the corresponding *Arabidopsis thaliana* ALS sequence). The constructed expression vector, referred to as PE2 (Fig. 1b), was introduced into embryogenic calli of peanut cultivar YuHua 9326 (YH9326) via microprojectile bombardment (Figures S1 and S2; Supplementary Methods). Following 3 weeks of selection on MS medium containing 20 mg/L hygromycin B, 7,008 independent T_0_ callus lines were obtained. To enrich for edited events at the *AhALS2* loci, these callus lines were transferred to MS medium supplemented with 0.2 μM bispyribac-sodium (BS), an ALS-targeting herbicide to which cells carrying the target W574L mutation are resistant (Butt et al. 2020). After 3 weeks, all 635 surviving lines were genotyped via Sanger sequencing, revealing 7 lines with the desired edits, each as a single heterozygous mutation at either the *AhALS2-A* locus or *AhALS2-B* locus (Fig. 1c; Table S1), corresponding to a 0.1% editing efficiency (Table 1). No unintended edits including indel, or pegRNA scaffold-derived base substitution at the target sites (Anzalone et al. 2019; Jiang et al. 2020), were detected (Fig. 1c).

**Figure 1:**
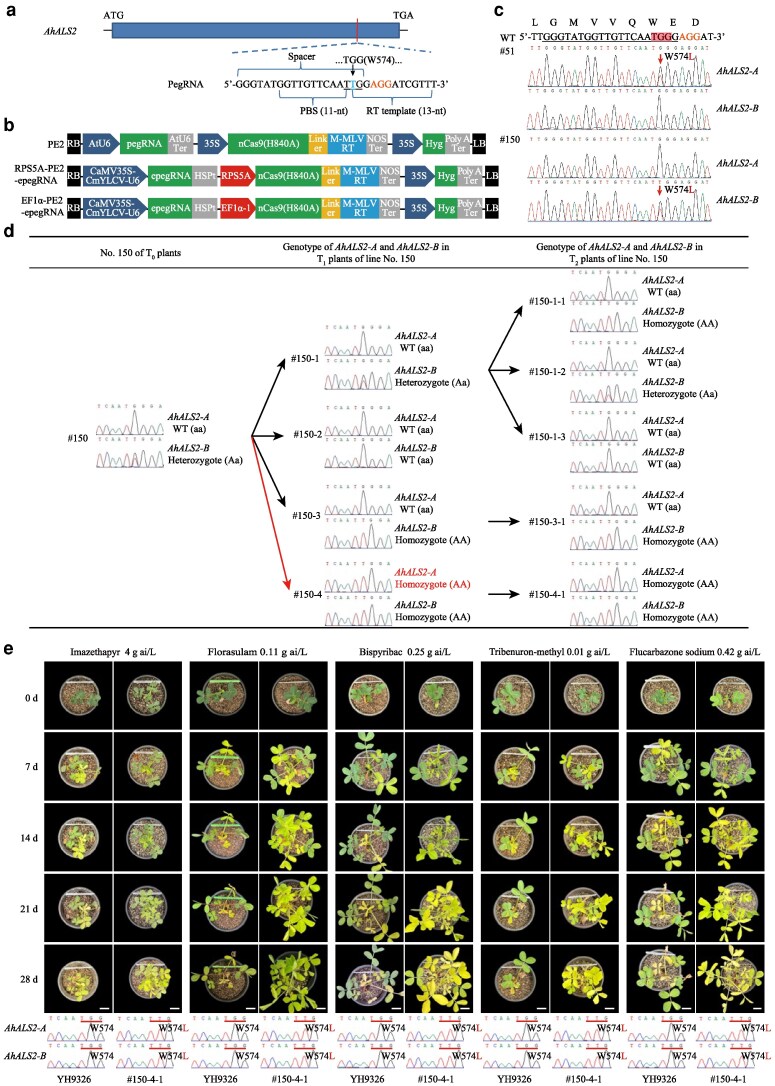
Prime editing-mediated base transversion at the W574 sites of *AhALS2* confers broad-spectrum herbicide resistance in peanut. **a)** Prime editing guide RNA (pegRNA) design for the W574L mutation at the *AhALS2* loci. The lengths of the primer binding site (PBS) sequence and reverse transcriptase (RT) template are indicated by the brackets. The protospacer adjacent motif (PAM) sequence and the target edit are highlighted in orange and blue, respectively. **b)** Schematic diagram of the T-DNA region of the prime editors used in this study. AtU6: *Arabidopsis* U6 promoter; AtU6 Ter, AtU6 terminator; 35S, 35S promoter of cauliflower mosaic virus (CaMV); nCas9 (H840A), Cas9 (H840A) nickase; M-MLV RT, Moloney murine leukemia virus RT; NOS Ter, nopaline synthase terminator; Hyg, hygromycin B resistance gene; CaMV35S-CmYLCY-U6, a composite promoter comprising of CaMV35S enhancer, promoter from Cestrum yellow leaf curling virus and shortened AtU6-26 promoter; epegRNA, pegRNA with the tevopreQ1 motif; HSPt, the heat shock protein 18.2 terminator from *Arabidopsis*; RPS5A, peanut ribosomal protein S5 promoter; EF1α−1, peanut elongation factor 1-alpha promoter; RB and LB represent the right and left T-DNA borders, respectively. **c)** Representative sequencing chromatograms of the prime-edited target sites of *AhALS2-A* and *AhALS2-B*. The PAM sequence is labeled in orange, while the target site is highlighted with a red box. Mutations are indicated by red arrows and red letters. WT represents the wild-type sequence. **d)** Genetic transmission of PE-mediated edits from the T_0_ to the T_2_ generation. The re-editing event and the resulting mutation are indicated in red. **e)** Herbicide resistance symptoms in WT YH9326 and T_2_ mutant plants of line #150-4-1 after treatment with 5 different ALS-inhibiting herbicides. Ai, active ingredient. Photographs taken at 0, 7, 14, 21 and 28 days after herbicide treatment are shown (labeled 0 d to 28 d on the left). Images were digitally extracted for comparison. The reading frame of the target site is underlined, with mutations indicated in red. Scale bar = 3 cm.

**Table 1:** Summary of prime editing efficiencies at the target AhALS2 loci.

Vector	Mutation	Transformed calli	Hygromycin-resistant events	Bispyribac-sodium resistant events	Sequenced events	Edited events	Editing efficiency^a^	Homozygous mutations
PE2	W574L (TGG-TTG)	64,800	7,008	635	635	7	0.10%	0
RPS5A-PE2-epegRNA	W574L (TGG-TTG)	16,200	1,188	5	5	1	0.08%	0
EF1α-PE2-epegRNA	W574L (TGG-TTG)	10,800	348	5	5	1	0.29%	0

^a^The editing efficiency was calculated by dividing the number of edited events by the number of hygromycin-resistant events.

T_0_ plants were successfully regenerated from 5 out of the 7 edited callus lines (Table S1) and cultivated in a greenhouse under a 16 h/8 h light/dark cycle at 28 ℃/25 ℃ for approximately 120 days. All regenerated lines produced seeds. Genotyping T_1_ plants from these 5 lines confirmed the successful transmission of the edits from T_0_ plants to the T_1_ generation. Notably, continued editing was observed in lines #150 and #177, resulting in the generation of 2 sublines, #150-4 and #177-3, with the W574L mutation at both the *AhALS2-A* and *AhALS2-B* loci (Fig. 1d; Figure S3). All mutant lines were advanced to the T_2_ generation. Progenies from lines #150-3 and #150-4, along with wild-type (WT) YH9326 plants, were tested for herbicide resistance (Figure S4; Supplementary Methods). Results demonstrated that plants carrying the W574L mutation exhibited substantially higher tolerance to all 5 types of ALS-targeting herbicides compared with YH9326 (Fig. 1e; Figures S5 and S6). An evaluation of major agronomic traits in the T_2_ double mutant line #150-4-1 and single mutant line #150-3-1 showed no substantial differences from YH9326 (Figures S7 and S8; Supplementary Methods). Off-target effect analysis in T_3_ progenies derived from lines #150-4-1 and #150-3-1 confirmed the absence of off-target editing events (Table S2 and Figure S9; Supplementary Methods). However, PCR-based transgene detection indicated that the tested T_3_ plants still retained vector backbone sequences (Figure S10, Table S3). Therefore, backcrossing with the WT YH9326 would still be necessary to generate nontransgenic, genome-edited lines suitable for breeding and downstream application.

The low editing efficiency of PE observed in this study (Table 1), which is consistent with findings in other dicot crop species (Lu et al. 2021; Perroud et al. 2022), considerably limits the broader application of this versatile tool for peanut improvement. To address this, we employed 2 strategies to improve the performance of PE2. The first strategy involved adding a structured RNA motif, tevopreQ1 (Nelson et al. 2022), to the 3′ terminus of pegRNA and placing it under the control of the composite CaMV35S-CmYLCV-U6 promoter (Jiang et al. 2020) to enhance both pegRNA expression and transcript stability (Text S2). The second strategy utilized 2 endogenous promoters (Lu et al. 2021), a peanut ribosomal protein S5 (RPS5A; LOC112728593) promoter and a peanut elongation factor 1-alpha (EFlα-1; LOC112712578) promoter (Text S3 and S4), to direct the expression of nCas9-M-MLV-RT fusion protein. The resulting vectors, designated as RPS5A-PE2-epegRNA and EF1α-PE2-epegRNA (Fig. 1b), were used for peanut transformation. Following 3 weeks of selection on hygromycin, 1,188 and 348 T_0_ callus lines were obtained for RPS5A-PE2-epegRNA and EF1α-PE2-epegRNA, respectively. After an additional 4 weeks of BS selection, 5 resistant lines were obtained from each transformation. Genotyping of the BS-resistant callus lines showed that both RPS5A-PE2-epegRNA and EF1α-PE2-epegRNA generated one edited line each with the desired G•C to T•A transversion at either the *AhALS2-A* or *AhALS2-B* locus. The corresponding editing efficiencies were 0.08% for RPS5A-PE2-epegRNA and 0.29% for EF1α-PE2-epegRNA (Table 1).

Compared with PE2, EF1α-PE2-epegRNA demonstrated a 2.9-fold increase in editing efficiency (0.29% vs. 0.1%), whereas RPS5A-PE2-epegRNA performed slightly less efficiently (0.08% vs. 0.1%). This contrast suggests that the improved performance of EF1α-PE2-pegRNA is more likely due to the use of the endogenous EFlα-1 promoter than to the pegRNA enhancement. If pegRNA modification were the dominant factor, both vectors would be expected to yield similar improvements. This interpretation aligns with previous findings that endogenous promoters outperform CaMV35S in promoting precise base editing and prime editing in dicot species such as *Arabidopsis* and tomato (Kang et al. 2018; Choi et al. 2021; Lu et al. 2021). Although pegRNA design remains a critical factor (Jiang et al. 2020; Kim et al. 2021; Feng et al. 2022; Nelson et al. 2022), our data indicate that, in this context, the low editing activity of the nCas9-RT fusion protein may be the primary bottleneck limiting editing outcomes. A recent study reported that markedly enhanced prime editing efficiency was achieved in tomato by optimizing both the PE protein architecture and pegRNA expression (Vu et al. 2024). Future efforts will focus on validating the advantage of peanut endogenous promoters for driving PE expression, further engineering the protein components of the PE system, and testing multiple target sites and diverse edit types in peanut to identify optimal PE architectures for more efficient genome editing in this crop.

In conclusion, we successfully generated herbicide-resistant alleles in peanut using prime editing. Optimization of the PE system, including the use of the peanut EF1α-1 promoter to drive nCas9-M-MLV-RT expression and the CaMV35S-CmYLCV-U6 composite promoter to express tevopreQ1-tagged pegRNA, resulted in a 2.9-fold increase in editing efficiency. These results provide a valuable foundation for advancing prime editing research and applications in this economically important oilseed crop.

## Supplementary Material

kiag116_Supplementary_Data

## Data Availability

The data underlying this article are available in the article and in its online supplementary material.
