# Supplementary material for: Development of herbicide-resistant peanut via prime editing and optimization of editing efficiency
Source: Plant Physiol. 2026 Mar 25;200(3):kiag116. doi: 10.1093/plphys/kiag116 (PMC13013096; doi:10.1093/plphys/kiag116)
Supplement: kiag116_Supplementary_Data [file kiag116_supplementary_data.pdf]

Supplementary Data

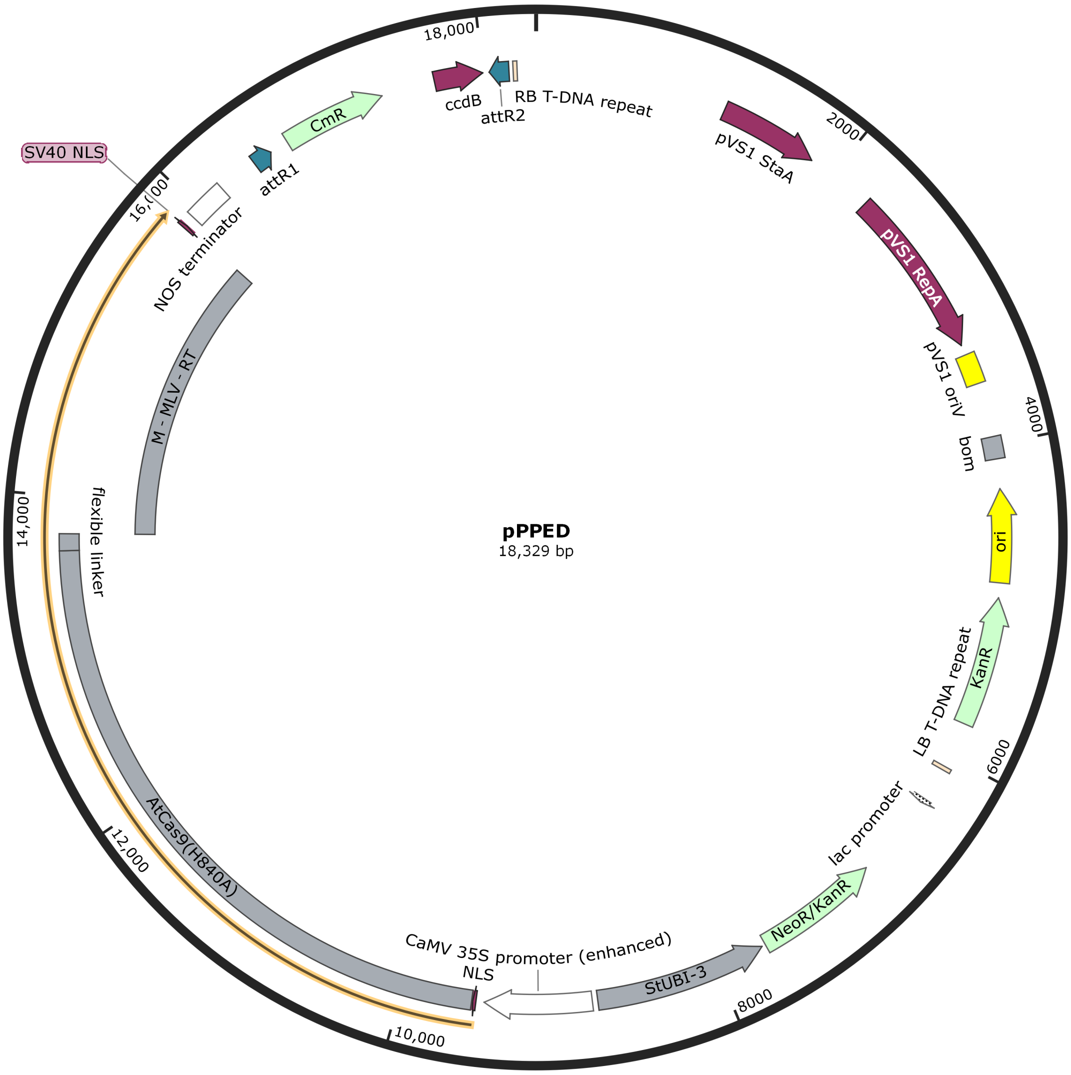

Figure S1. Plasmid map of the pPPED vector. CaMV 35S promoter, 35S promoter of cauliflower mosaic virus; NLS, nuclear localization signal; AtCas9(H840A), Arabidopsis codon-optimized Cas9 nickase; M-MLV-RT, Moloney murine leukemia virus reverse transcriptase; NOS terminator, nopaline synthase terminator.

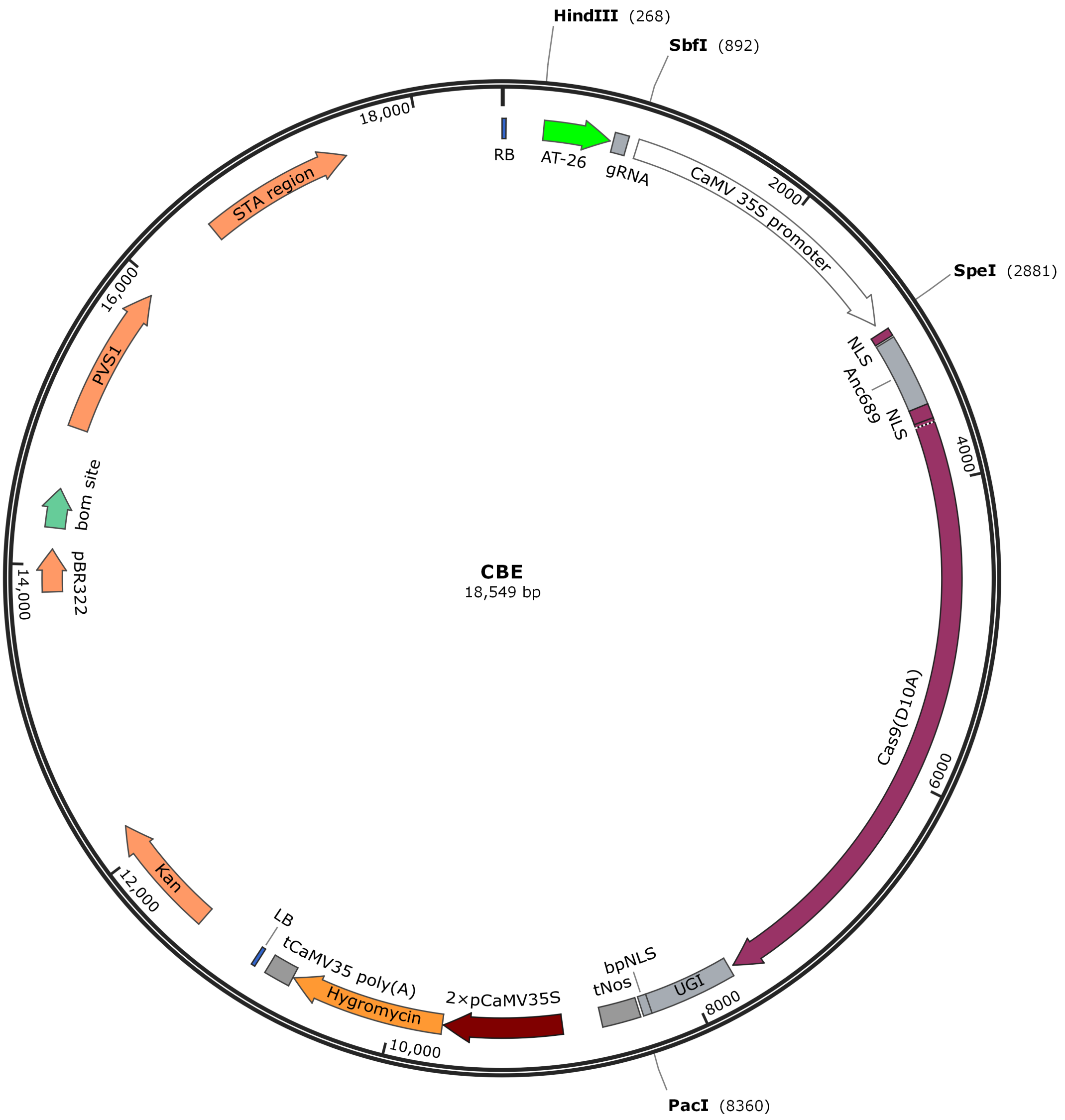

Figure S2. Plasmid map of the cytosine base editor construct used to generate PE2, RPS5A-PE2-epegRNA, and EF1α-PE2-epegRNA vectors. AT-26: Arabidopsis U6-26 promoter; gRNA, guide RNA; CaMV 35S promoter, 35S promoter of cauliflower mosaic virus; NLS, nuclear localization signal; Anc689, a cytidine deaminase; Cas9(D10A): Cas9 nickase; UGI: uracil-DNA glycosylase inhibitor; bpNLS, bipartite NLS; tNos, nopaline synthase terminator; RB and LB represent the right and left T-DNA borders, respectively.

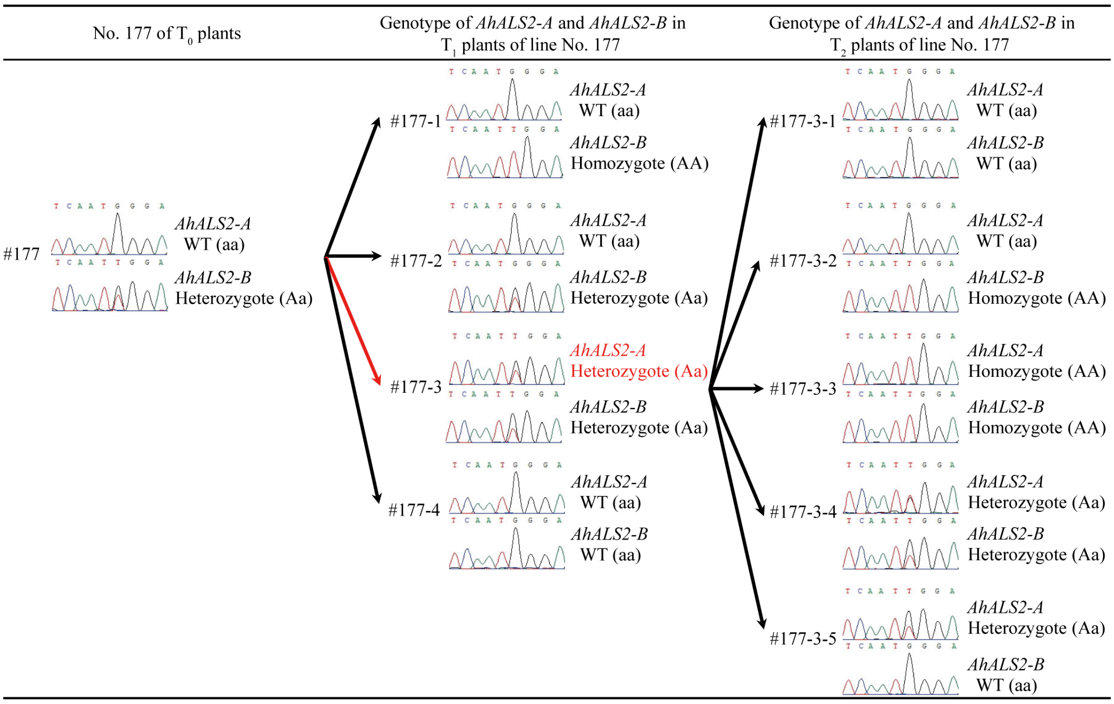

Figure S3. Genetic transmission of PE-mediated edits from T_0 to T_2 generation. The re-editing event and the resulting mutation are indicated in red.

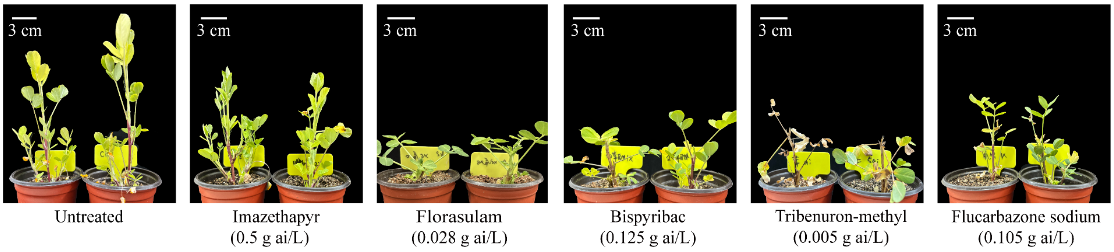

Figure S4. Determination of the minimum inhibitory concentration of five herbicides on wild-type YH9326 seedlings. Ai, active ingredient. Seedlings at the 3- to 6-leaf stage were treated with a single application of the respective herbicides. Plant phenotypes were assessed and photographed at 21 days after treatment. Images were digitally extracted for comparison. All treatments were performed with three independent biological replicates.

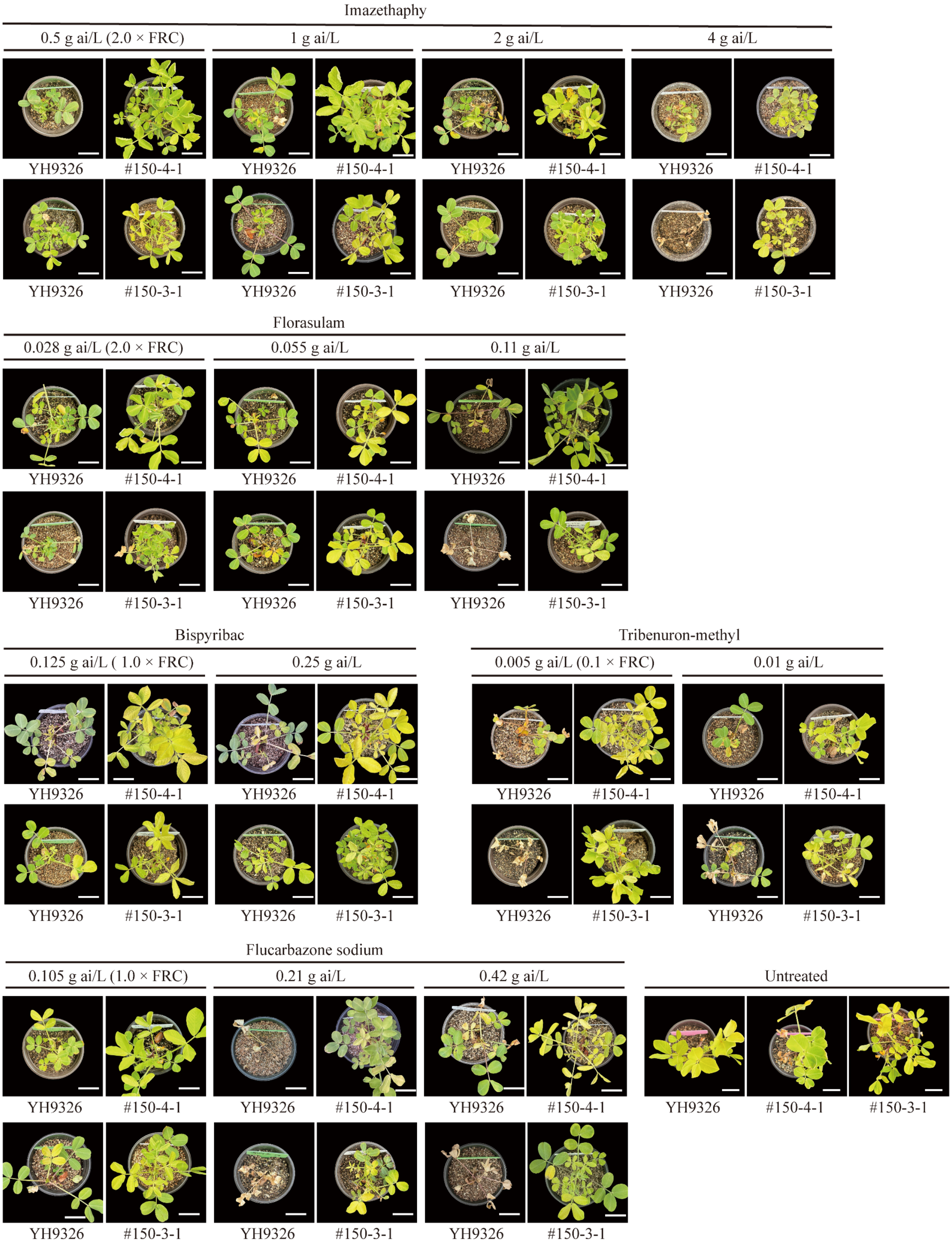

Figure S5. Herbicide resistance symptoms in wild-type YH9326 and T_2 mutant plants of lines #150-4-1 and #150-3-1. Ai, active ingredient. FRC, field recommended concentration. Pictures were taken 28 days after herbicide treatment. Images were digitally extracted for comparison. Scale bar = 3 cm.

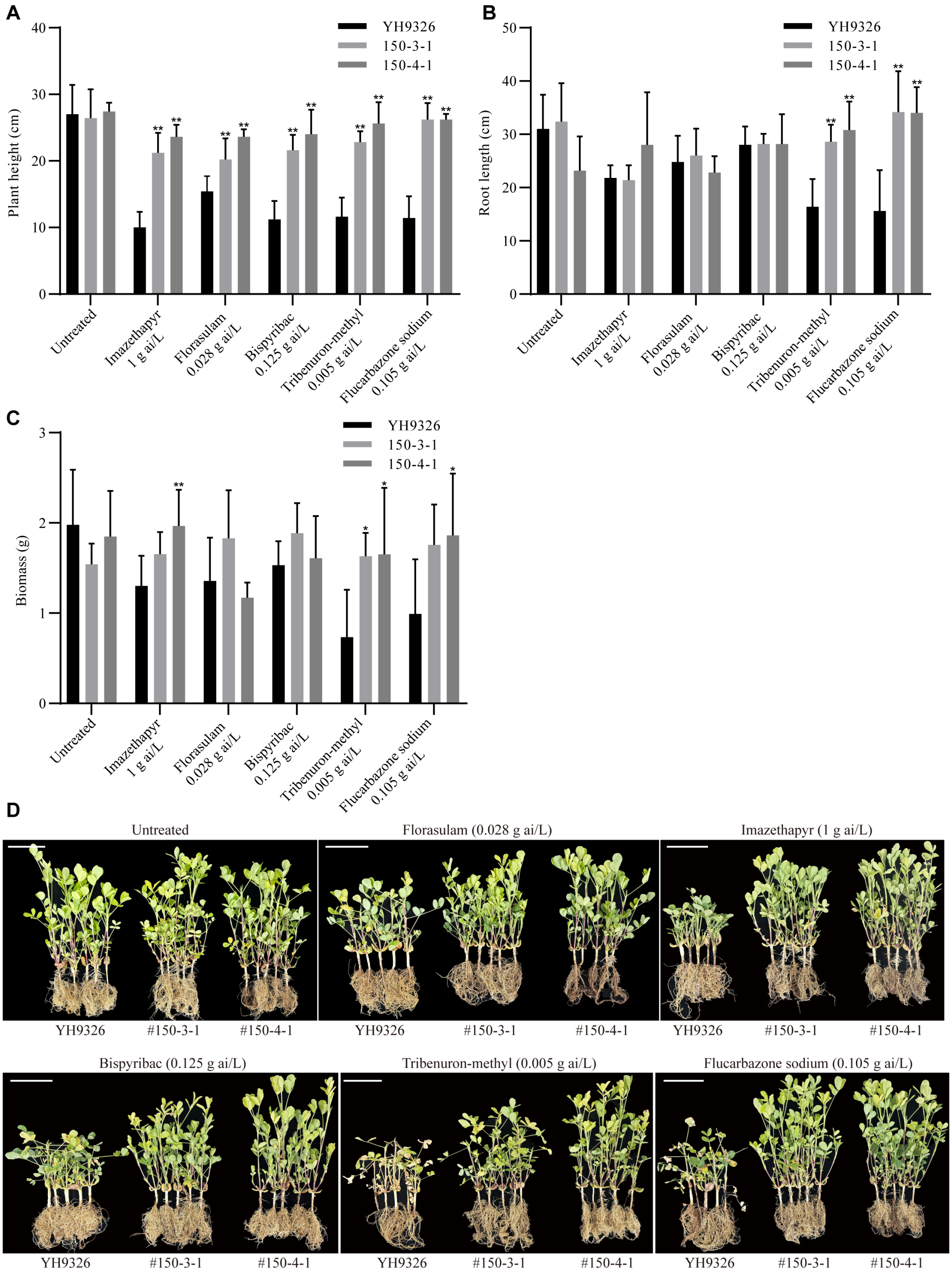

Figure S6. Phenotypic analysis of wild-type (WT) and mutant lines after herbicide treatment. **(A-C)** Plant height (A), root length (B), and dry biomass (C) of WT and mutant lines #150-3-1 and #150-4-1 following herbicide application. Ai, active ingredient. Data are presented as mean ± SD (n = 5). Statistical significance was analyzed by one-way analysis of variance followed by Fisher's LSD post-hoc test; asterisks indicate significant differences compared to the WT control (**P* < 0.05, ***P* < 0.01). **(D)** Phenotypes of all five biological replicate plants per line, photographed at 28 days post herbicide treatment. Images were digitally extracted for comparison. Scale bar = 10 cm.

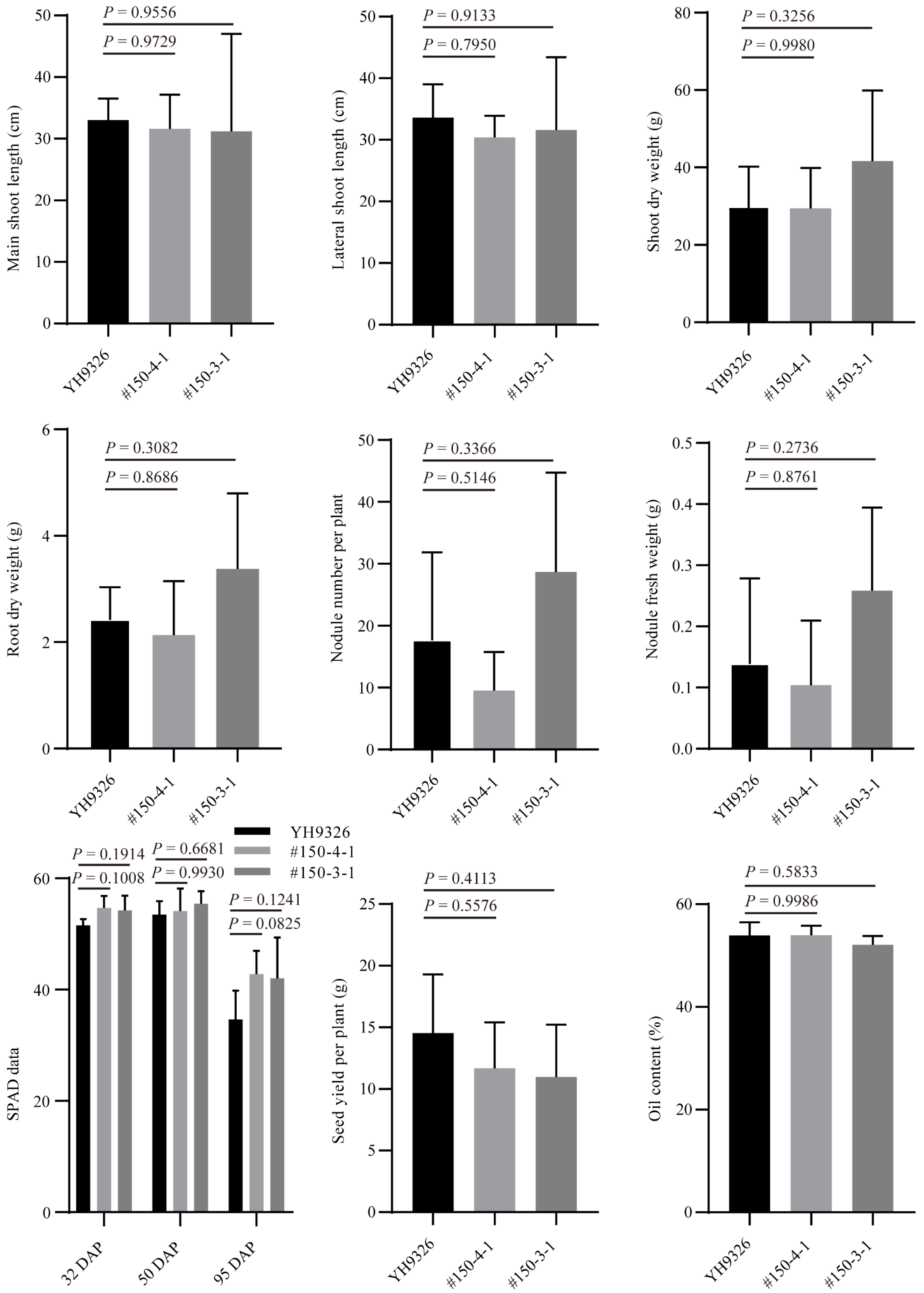

Figure S7. Investigation of major agronomic traits in wild-type YH9326, mutant lines #150-4-1 and #150-3-1. DAP, days after planting. Chlorophyll content was measured with a handheld SPAD-502Plus chlorophyll meter (SPAD, Soil Plant Analysis Development), and other agronomic traits were assessed at plant maturity (120 DAP). Data are shown as mean ± SD (n ≥ 3). *P*-values were calculated using a one-way analysis of variance test.

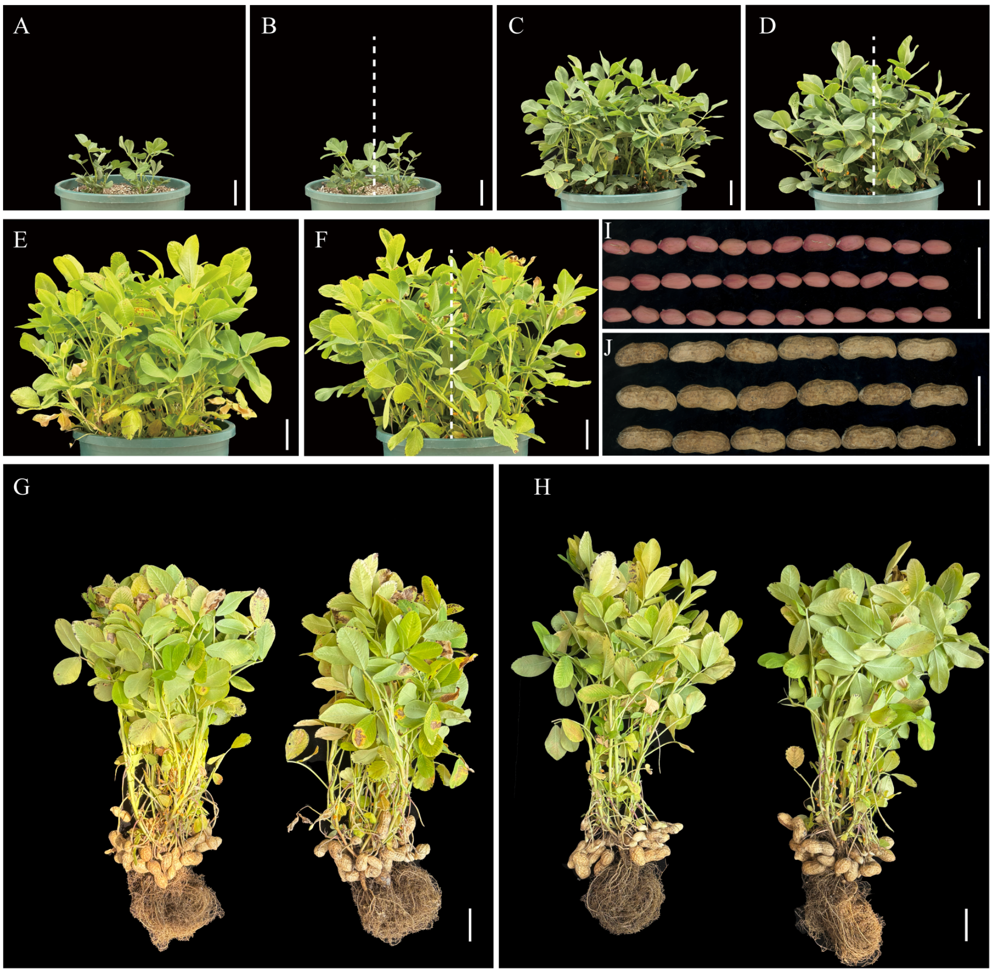

Figure S8. Phenotypic comparison of the wild-type YH9326 and mutant lines #150-4-1 and #150-3-1 at different developmental stages. (A) YH9326 at 21 days after planting (DAP). (B) Mutant lines #150-4-1 (left of the dashed line) and #150-3-1 (right of the dashed line) at 21 DAP. (C) YH9326 at 59 DAP. (D) Mutant lines #150-4-1 (left of the dashed line) and #150-3-1 (right of the dashed line) at 59 DAP. (E) YH9326 at 94 DAP. (F) Mutant lines #150-4-1 (left of the dashed line) and #150-3-1 (right of the dashed line) at 94 DAP. (G) Harvested plants of YH9326 (left) and line #150-4-1 (right). (H) Harvested plants of YH9326 (left) and line #150-3-1 (right). (I) Seeds of YH9326 (bottom), lines #150-4-1 (middle), and #150-3-1 (top). (J) Kernels of YH9326 (bottom), lines #150-4-1 (middle), and #150-3-1 (top). Images were digitally extracted for comparison. Scale bar = 5 cm.

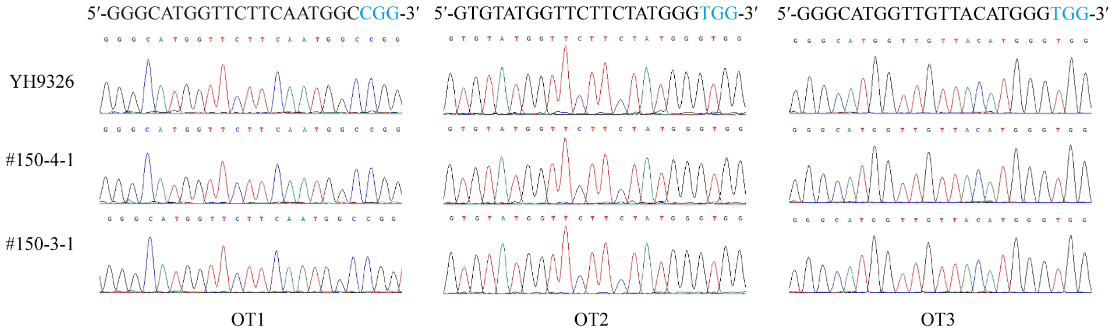

Figure S9. Representative Sanger sequencing chromatograms of the off-target sites in edited lines. The protospacer adjacent motif sequences are highlighted in blue. OT1-3, predicted off-target sites.

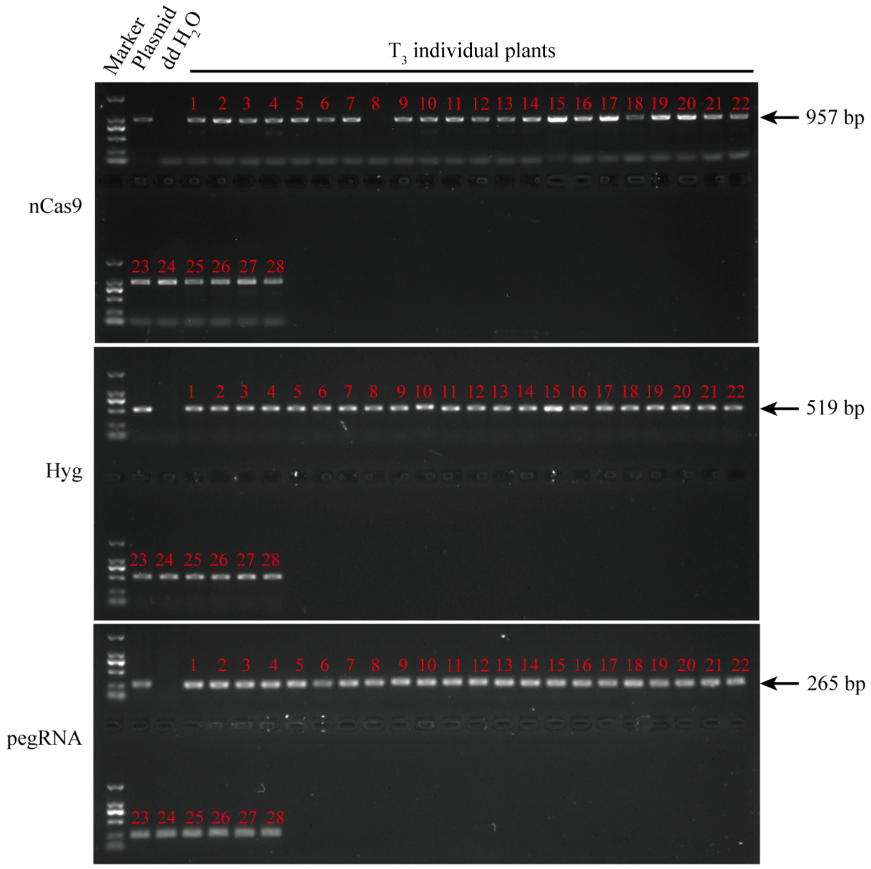

Figure S10. Detection of transgenes in edited T_3 plants derived from lines #150-4-1 (plants 1-16) and #150-3-1 (plants 17-28). nCas9, Cas9(H840A) nickase; Hyg, hygromycin B resistance gene; pegRNA, prime editing guide RNA.

Table S1. Mutation analysis of the edited lines generated by prime editing

Line	*AhALS2-A*	*AhALS2-B*
#51[a]	W574L (He)	WT
#150	WT	W574L (He)
#165	W574L (He)	WT
#177	WT	W574L (He)
#362[a]	WT	W574L (He)
#545	WT	W574L (He)
#556	W574L (He)	WT

[a] Regeneration of T_0 plants from these lines has failed. He, heterozygous. WT, wild-type.

Table S2. Off-target analysis in T_3 plants of lines #150-4-1 and #150-3-1

Off-target site	Putative off-target sequences (5′-3′)[a]	Nucleotide mismatch number	Chromosome position	Number of tested plants	Number of plants with off-target editing
OT1	GGGcATGGTTcTTCAATGGcCGG	3	Arahy.02: 84831251..84831272; Arahy.12: 98435417..98435439	28	0
OT2	GtGTATGGTTcTTCtATGGGTGG	3	Arahy.06: 43820006..43820028	28	0
OT3	GGGcATGGTTGTTacATGGGTGG	3	Arahy.13: 28606041..28606063	28	0

[a] Lowercase letters indicate mismatched bases.

Table S3. Primers used in this study

Primer	Sequence (5′ to 3′)	Purpose
hr-PE-M-F	catttacgaacgataactagtATGGCACCTAAGAAAAAGAGGAAG	For amplifying the nCas9-M-MLV-RT coding sequence
hr-PE-M-R	aacgatcggggaaatttaattaaTCACACCTTCCTCTTCTTCTTGG	
hr-pe-EF1α-F	ctgcagacaaatggccctgcaggGAAAAATCTGACATGGCTCTC	For amplifying the EF1α-1 promoter
hr-pe-EF1α-R	tttcttaggtgccatactagtGATTTCTTAAAACCTTAAACAAAG	
hr-pe-RPS5A-F	ctgcagacaaatggccctgcaggGAATATGTCTAAAGTTGTTGCTT	For amplifying the RPS5A promoter
hr-pe-RPS5A-R	tttcttaggtgccatactagtTCCTGCAAACGAGAATTGAAC	
ALS10F	GGCGGCATATGAATGCTCTT	For amplifying the *AhALS2-A* target site
ALS10R	AACCATTCCACAAGTGGCTGTA	
ALS20F	ATGCGTACTAACTCAACATATGG	For amplifying the *AhALS2-B* target site
ALS20R	ACAGACTAAGAATTAATCCGTGAC	
peCas9-F	CGCATCTCTCGGAACCTACC	For detecting nCas9 fragment in edited lines
peCas9-R	CCTCCCCTCTCAGCCTTAGT	
HYG-F	TTGGCGACCTCGTATTGGGAA	For detecting the hygromycin resistance gene fragment in edited lines
HYG-R	CAAAGATCGTTATGTTTATCGGCACT	
PegRNA-F	CAAAAGGCCCCTGGGAATCT	For detecting the pegRNA fragment in edited lines
PegRNA-R	CGACTCGGTGCCACTTTTTC	
OT1-02F	CCAAGATAACTATCATGCGGTTCAT	For amplifying off-target site OT1 on chromosome 02
OT1-02R	AGCATAGTTGAGATGCAAGAACAT	
OT1-12F	GTTATAGCTCGGCTGCCGTT	For amplifying off-target site OT1 on chromosome 12
OT1-12R	TGACGTGTGGGAAAAGCGAA	
OT2-F	GTTTAGTTTGATGCTTGATGGCTCT	For amplifying off-target site OT2
OT2-R	TCAGCCTATTGCGTCCACCAGTGT	
OT3-F	TTAGGAAGGCTTGTTCCCCCTT	For amplifying off-target site OT3
OT3-R	TTTGTCACCCCTCCAGGTCA	

Supplementary Text S1

AtU6 promoter-target-gRNA scaffold-RTT-PBS-AtU6 terminator

CATTCGGAGTTTTTGTATCTTGTTTCATAGTTTGTCCCAGGATTAGAATGATTAGGCATC
GAACCTTCAAGAATTTGATTGAATAAAACATCTTCATTCTTAAGATATGAAGATAATCTT
CAAAAGGCCCCTGGGAATCTGAAAGAAGAGAAGCAGGCCCATTTATATGGGAAAGAA
CAATAGTATTTCTTATATAGGCCCATTTAAGTTGAAAACAATCTTCAAAAGTCCCACATC
GCTTAGATAAGAAAACGAAGCTGAGTTTATATACAGCTAGAGTCGAAGTAGTGATTGG
GTATGGTTGTTCAATGGGGTTTTAGAGCTAGAAATAGCAAGTTAAAATAAGGCTAGTCC
GTTATCAACTTGAAAAAGTGGCACCGAGTCGGTGCAAACGATCCTCCAATTGAACAAC
CTTTTTTTTGCAAAATTTTCCAGATCGATTTCTTCTTCCTCTGTTCTTCGGCGTTCAATTT
CTGGGGTTTTCTCTTCGTTTTCTGTAACTGAAACCTAAAATTTGACCTAAAAAAAATCT
CAAATAATATGATTCAGTGGTTTTGTACTTTTCAGTTAGTTGAGTTTTGCAGTTCCGATG
AGATAAACCAATA

Supplementary Text S2

CaMV35S-CmYLCV-U6 promoter-tRNA-pegRNA-linker-tevopreQ1-HDV-polyT-HSPt

ATGGAGTCAAAGATTCAAATAGAGGACCTAACAGAACTCGCCGTAAAGACTGGCGAA
CAGTTCATACAGAGTCTCTTACGACTCAATGACAAGAAGAAAATCTTCGTCAACATGG
TGGAGCACGACACACTTGTCTACTCCAAAAATATCAAAGATACAGTCTCAGAAGACCA
AAGGGCAATTGAGACTTTTCAACAAAGGGTAATATCCGGAAACCTCCTCGGATTCCATT
GCCCAGCTATCTGTCACTTTATTGTGAAGATAGTGGAAAAGGAAGGTGGCTCCTACAA
ATGCCATCATTGCGATAAAGGAAAGGCCATCGTTGAAGATGCCTCTGCCGACAGTGGT
CCCAAAGATGGACCCCCACCCACGAGGAGCATCGTGGAAAAAGAAGACGTTCCAACC
ACGTCTTCAAAGCAAGTGGATTGATGTGATTGGCAGACATACTGTCCCACAAATGAAG
ATGGAATCTGTAAAAGAAAACGCGTGAAATAATGCGTCTGACAAAGGTTAGGTCGGCT
GCCTTTAATCAATACCAAAGTGGTCCCTACCACGATGGAAAAACTGTGCAGTCGGTTT
GGCTTTTTCTGACGAACAAATAAGATTCGTGGCCGACAGGTGGGGGTCCACCATGTGA
AGGCATCTTCAGACTCCAATAATGGAGCAATGACGTAAGGGCTTACGAAATAAGTAAG
GGTAGTTTGGGAAATGTCCACTCACCCGTCAGTCTATAAATACTTAGCCCCTCCCTCATT
GTTAAGGGAGCAAAATCTCAGAGAGATAGTCCTAGAGAGAGAAAGAGAGCAAGTAGC
CTAGAAGTAGTCAAGGCGGCGAAGTATTCAGGCACGTGGCCAGGAAGAAGAAAAGCC
AAGACGACGAAAACAGGTAAGAGCTAAGCATCTAGAAAGTTGAAAACAATCTTCAAA
AGTCCCACATCGCTTAGATAAGAAAACGAAGCTGAGTTTATATACAGCTAGAGTCGAA
GTAGTGATTGAACAAAGCACCAGTGGTCTAGTGGTAGAATAGTACCCTGCCACGGTAC
AGACCCGGGTTCGATTCCCGGCTGGTGCAGGGTATGGTTGTTCAATGGGGTTTTAGAG
CTAGAAATAGCAAGTTAAAATAAGGCTAGTCCGTTATCAACTTGAAAAAGTGGCACCG
AGTCGGTGCAAACGATCCTCCAATTGAACAACCCGGAAGAACGCGGTTCTATCTAGTT
ACGCGTTAAACCAACTAGAAGGCCGGCATGGTCCCAGCCTCCTCGCTGGCGCCGGCTG
GGCAACATGCTTCGGCATGGCGAATGGGACTTTTTTTTGATATCTCCGGGGCTAATTGA
ATATGAAGATGAAGATGAAATATTTGGTGTGTCAAATAAAAAGCTGGTGTGCTTAAGTT
TGTGTTTTTTTCTTGGCTTGTTGTGTTATGAATTTGTGGCTTTTTCTAATATTAAATGAAT
GTAAGATCTCATTATAATGAATAAACAAATGTTTCTATAATCCATTGTGAATGTTTTGTTG

GATCTCTTCTGCAGCATATAACTACTGTATGTGCTATGGTATGGACTATGGAATATGATTA
AAGATAAG

Supplementary Text S3

Peanut RPS5A promoter

GAATATGTCTAAAGTTGTTGCTTTTTATCTATATATATTTACTTCTATGCATAAATTTTTATT
CTCTTGCTATTCAAGACAGTTGTTTCTTCTTCAATTTTGGACAGCTAAACTGAGTTTATA
TGAATTGATGTCCTTCTTTTGTTATATTTTGTTTGTTTTCAGGACAAAGAGATCGATACA
AGTATAATCACAAACTACCTGGTTCTTCAACATGATTTGTTCCACATGTTTAGACAAAG
CAAGATGTTTATGAGTTTTGACAATGTTATTACTTCAGCGAGTTATATTCCAAACTTATTT
TATGAAAATGGAGCAAAGTAGCTTATAAATACTATGGTAGGATCATATTTATAATACTGTC
CTGTGAAAATTCATTATTAGTTCATTATGGTTCTTGCTCTTTGGCGATTCTTCCCTTCTTG
TTTGTATAGGGACATTGCATTGCAAATAATGGACACTCACTCTCATGGAGAATAATACTC
TCATCATCACTGTGATTGCTTGCTTGCTTCCTTCCTTTCTTCCAAAGAAACAATTTAAGA
AGCCTATTTTCAAGGCATTGAAGTTTCCAATTGAACAATGATGTAATTTGGAACTTTTGT
TGACTGAGTTCTGTAGTTGAATGCAATCTATCTATTCATTATGTAAAATTGAGTTGGACA
TGAACCGTAAGAAATAAGTGGTCTGAGAGTGTAAAATGCTATTGTAGAGATAACATGA
ATAAATTTTGGTCTTTGGAGTATTGTTTCTTTCAGTATGATTCTTGTGTGCAATTGCAATC
ATGCTCTTTATTTTCAATTTTGCAATTTGCACTAAAACGAGTAAGGCTTTATCTAATGATA
AACTCAGATATATTTGGTCTCTTTTTTTTTTTTTTTTGTGTGGCTATATTTGGTATCTGTTT
GAAAGCCTTAATCGTTCTCTGAAAAAATTAATCCTTTTATAAAATAAATTATTTATTATCA
TTTTAATATTTTACTTTAATTTCCATAAATTAAATACTTGACAAACTAGATTCTTATGTAGT
AAACACAGTAAGTTAATTTCTAACCGTACTTACATAAAAGTATTTTATTGTATCCATACAT
GTGTAAAAGGTTTAATTACTCTATTGAATATTGATTCCTATAGTTTTGCAAAATTTTAATT
AGGTCTCTGTACTTTTTCTCCTTTTAATTAAGTCTTTGCACTAAAAATTTTTTTAATTAGG
TCTTTACACTTTTTTTCTTTTTTATTTGGATCTTTGTATCAAATTTTTTTTAGTTGGATCCT
TATAAAATTAAGCCAATGATTGTCAGGAAGAACCTAATTGAAAAAAAAAGTTAGTGCA
GAGAGACTCAATTAAAGAAAAAAAAAATATAGGGATCTAATTGAAAATTAAATCTATGT
AATTAAATCTATGTAAAATAATCAATAGAATAATTAAATCTATGTAAAATTTTACTTCTTA
ATTTCTATGCCTACAATGTTAATTTCTATGATAATCTATAGCCATAAGACTCATTTTTATTT
TTTTCAAGCTAAAAATTTTTACACTTTTTTTTTAAAAGAAAAATCAATTGAAAAAAAAA
AAGGTTTCCTACAAGCTGGTTGAGCTAATTAACCATGAGTCCATAAGATGATGCTTTTT
TTTTTTTTTTACTTGTTTCCTTCTGTTTTTGGTTATCCTTCCTTTCCCCTTTTGTTTTGGTC
ATGGTCTGGGTTTATTTTATTTAACCAACCTTACAATAATTCAAGAAGACTGGGCCAGTC
AACATGGTCTACAATTCTAATTACGGTTCATTTCCAAGCAAAGCCTCTTAGCTTAGGGC
TCCTTTGACTGATCCCAAAAATCAGGGTGCTTTCTAAAGTCCAATAAAAAAAAACCACA
AAAAACAGAAAAGAAATTAGGGTTCCTTTCTGATCAAGGTATAAATAACGAAATTCCA
AAACCGGCCCCAAGTAGGGTTTTCGCTTCACGAAGTGTGTTCGACTAATCCCTTATACT
CCACAGCATAGAACCCTAAGCGGCACAACACACTTTCTGCTTCAACAAGTTCGTCTCC
GTCTTCTTCTTTCTTCGATCCGTATCTCTTTTCTCTTTCAATCTTTACACTTAGCTAACTAT
CTATTCTGTGTTCAATTCTCGTTTGCAGGA

Supplementary Text S4

Peanut EF1α-1 promoter

GAAAAATCTGACATGGCTCTCGTAATACTTGTCACGCGTACACGTGTACACGTGGGTCA
CGTATACGCGTCGCCGGACAGACTTCCCTCTCACGCGTACGCGACGCATCGCCATAAAT
TCAACAAATTCTTATTTTTTTCATGAATTCTCCATTTTACATGCTTTTTTCTATTTCTTTCA
AACCATTTTTGCCTTCAGGGTCTATCTCATTTACACTGTAAATAAAATACATTTACACTG
TAAATGAGATAACCACGATGCAGCTGATATGTCATATCTCGTTTAAACTGTAAACGAGAT
ATGATTGAAAACGCAAAACGATAAATATTTTTAAAATTATTTATTTCCGTAATTAATGCTT
TTTTGTATTTATTTAAATAAAAAATCTTAGTTTACAGATGAGTTTTGATTTTTTTTTTCCC
TAATGTTCCTAGAAAAAATAGGATTTTAAGAGGTTAAAGATGAGCATAGAAGGATAATT
TAAAAATTTAAAGTGTTTTTAATAGAATTAATAAAAATCTACAATGAGATATTTTTATCTT
TTATAGGTATACTATTAGTTTAGTTTTTTTATGGTTAATTTTAAAACATTTAAATCTTTGAT
GAGTAGAAACTATAATTTACTCCTTTGTAAAAATAACAAAAATCAGTTGAGTGTTTACT
CTTTTATTTTACACAAATAAATTATATATCTATAAATTAATTATTGAATTAAGGTAACAATT
CAGGTGCAGTCGATTTTACGTGAAGTTGATATTGAGAGCCATTAGATAATTTAACTGATT
TTGACTAAATTTTTATCTAAGGCTCTCAGATATCAATTTCACGTAAAGTCTACTTCACTT
GAATTTTCACCTTAAATTAATTATTATATATTTATATATATTTATACATATTAATTTATATATAT
TATTTTGTTAAATAATTAACTGTGAGAATAATCAAATCTCTAATAACCAAATAATCAAAA
TTATTTACAGATTATAATTTTTTTAAAATTGCAAATACATTTTTATGCATGATGACTAAGTA
GTGGTTATTTTTTCTTGTATATATATATAACGTGACAATTTTATAATACTACTGTTTATATAT
CTTATATTTTTTAAAATAAATAATAACTAAAAAAACTAAAAAATTAGTACTTTTCGTAAA
AACTAAAGAATTAGCTAATATTTACTCAAATATAAAAAATATTAGTCATGCACATAAATG
AAAATATTAAATGGATCCAACAACCCTAAAAAGCAGCCCAATCGGATTTGAATCAACCC
GCTAACCCGAAAATCCGAAATAGCTAAAAACTTGTGTTGTGCCGCCGCATTATAATGCA
ACAAACGAGACGAGTTGTTGGCGCAACGCTGGGGTTCGAATCTTCGCGGATAAGCAC
ATCAAACGGACACAAATGGAACGTGGTCAATAATCTGAGGGCAATTCCGTCAAAGTAA
AATATCGAATTCAGCTATATAAACACTCTTAACCCTATCAAACTCGCACACTCTGCTCCC
GTACACTCTCCACGCTCACTTGCGGCTAGGGTTTTAGCTCAGCTCCTTCTTCCAGGTT
CGTTCCTTTGTTTTTCTTTCTCTCTTTCCGTTCCTTTGATTCGTCGTTGTTGTGTTGTGAT
AATCCATGCAATAGTTTATTTTTCGATCTATGTTTTCAGCAAATTATAGTTTTCCGATATTT
CTTGGAGTACTTTTCTTCAGTATAAATGTTTTCTTCGATTATTCTGGTTTTCTTGATTTTT
ATGTTGAATATTTGAATTGATTTGGTTCTTGTTTCGATTTATGAGCATGTTTAATTGAAAA
ATAGATCCAGAATTTTGAAAAATCGCATGTAATTTTGAATCTGGATATTCTCCTTTTTATC
TATGTATAGATCTGGGGGATTTGGTTTGAATATGGTTCACAATGATTTGCTAAAAAGGGT
AATTATTGGTTCGTGGTAATATTCGTTCGATCTGTTGTGACGTATCTCTTTGCTTTTGTGT
CTTGAACATTTCAAAAGCGTTAATCTCTGCATATTTTGATCAAAACTTATTATTTGATTTG
CGAGTATGTATTGTAATTTTGTATGCGAGTAAGATTGTTCTGAATGCGAATCTCACGGGT
CTATTTTATTTGGCAGATCTTTGTTTAAGGTTTTAAGAAATC

Methods

Plant materials and growth conditions

The wild-type (WT) peanut cultivar YuHua9326 (YH9326), bred by the Henan Academy of Agricultural Sciences, was used in this study. Plants were grown in the field located in Xinxiang, Henan province, China (35.02° N, 113.71° E). Seeds harvested from WT plants were used for callus induction and subsequent peanut transformation. Both edited T_0 lines and their progeny were cultivated in plant pots containing approximately 15 L of fresh, nutrient-rich potting soil (Zhengzhou NongLe Seedling Matrix Processing Factory, Zhengzhou, Henan, China). The soil composition included ≥ 3% nitrogen, phosphorus, and potassium, > 45% organic matter, and ≥ 0.2% trace elements. Two seedlings or seeds were planted per pot. Plants were grown under controlled greenhouse conditions with a 16-h light (28 °C)/8-h dark (25 °C) cycle and 50% relative humidity. The total growth period was 120 days. WT YH9326 plants were included as controls in all experiments to allow for comparative phenotypic and molecular analyses.

Vector construction and peanut transformation

The expression vector for dicot plant prime editing, pPPED (Figure S1) was a gift from Adam Bogdanove (Addgene plasmid # 162468; http://n2t.net/addgene:162468; RRID:Addgene_162468) (Wang et al., 2021). A cytosine base editor (CBE) preserved in our lab, containing multiple unique restriction sites for convenient replacement of vector components (Figure S2), was used to construct PE2, RPS5A-PE2-epegRNA, and EF1α-PE2-epegRNA vectors. First, the CBE was digested with *Spe* I and *Pac* I (ThermoFisher, Waltham, MA, USA) to remove the cytosine deaminase-nCas9 fusion protein. Next, PrimeSTAR GXL DNA Polymerase (Takara, Tokyo, Japan) and primers hr-PE-M-F and hr-PE-M-R were used to amplify the nCas9(H840A)-M-MLV-RT (Cas9(H840A) nickase-Moloney murine leukemia virus reverse transcriptase) fragment from pPPED as a template. The 50 μL PCR reaction, which was used for all subsequent PCRs in this study, contained 100 ng of DNA template, 1× PrimeSTAR GXL Buffer, 200 μM of each dNTP, 0.3 μM of each primer, and 1.25 U of PrimeSTAR GXL DNA Polymerase. The amplification protocol included an initial denaturation at 94 °C for 30 s, followed by 30 cycles of 98 °C for 10 s, 60 °C for 15 s, and 68 °C for 6.5 min, with a final extension at 72 °C for 10 min. The resulting PCR product was purified and cloned into the linearized CBE vector using an In-Fusion Snap Assembly cloning kit (Takara). The resulting construct was then digested with *Hin*d III and *Sbf* I (ThermoFisher) to incorporate the

prime editing guide RNA (pegRNA) module. The pegRNA for the PE2 vector was designed using the online tool PlantPegDesigner (http://www.plantgenomeediting.net/) (Lin et al., 2021), and the AtU6 promoter-pegRNA-AtU6 terminator fragment was synthesized by BGI Genomics (Shenzhen, China). This pegRNA expression cassette was ligated into the linearized vector using T4 DNA ligase (Vazyme, Nanjing, China), yielding the PE2 vector.

To construct RPS5A-PE2-epegRNA and EF1α-PE2-epegRNA, the PE2 vector was first digested with *Sbf* I and *Spe* I (ThermoFisher) to remove the CaMV35S promoter driving the nCas9-M-MLV-RT fragment. Next, the promoters of RPS5A and EF1α-1 were amplified using PrimeSTAR GXL DNA Polymerase (Takara) with primer pairs hr-pe-RPS5A-F/R and hr-pe-EF1α-F/R, respectively. The amplification protocol consisted of an initial denaturation at 94 °C for 30 s, followed by 30 cycles of 98 °C for 10 s, 55 °C for 15 s, and 68 °C for 2 min, with a final extension at 72 °C for 5 min. The PCR products were introduced into the linearized vector using the In-Fusion Snap Assembly cloning kit (Takara), resulting in RPS5A-PE2 and EF1α-PE2 constructs, respectively. Subsequently, both vectors were respectively digested with *Hin*d III and *Sbf* I (ThermoFisher) to add the engineered pegRNA module. The same sgRNA, primer binding site sequence, and reverse transcriptase template used in PE2 were applied to RPS5A-PE2 and EF1α-PE2. The linker sequence between the pegRNA and the tevopreQ1 motif was designed using the online tool pegLIT (https://peglit.liugroup.us/) (Nelson et al., 2022). The CaMV35S-CmYLCV-U6-pegRNA-linker-tevopreQ1-polyT-HSPt fragment was synthesized by BGI Genomics (Shenzhen, China), and ligated into the linearized RPS5A-PE2 and EF1α-PE2 vectors using T4 DNA ligase (Vazyme), creating RPS5A-PE2-epegRNA and EF1α-PE2-epegRNA, respectively.

Constructed expression vectors were transformed into peanut calli via microprojectile bombardment using a Scientz GJ-1000 helium-driven particle delivery system (Ningbo Scientz Biotechnology Co., Ltd, Ningbo, Zhejiang, China), following the manufacturers' protocol. Transformed calli were cultured on Murashige and Skoog (MS) medium (Murashige and Skoog, 1962) supplemented with 33 mg/L glutamine and 20 mg/L hygromycin and incubated at 28 °C in darkness. Following primary selection, hygromycin-resistant callus lines were transferred to a secondary selection medium containing 0.2 μM bispyribac-sodium and maintained for an additional three to four weeks. Surviving calli were collected for genotyping to confirm successful genome editing. Confirmed edited calli were then subject to root and shoot induction as previously described

(Joshi et al., 2005). Regenerated seedlings approximately 10 cm in height were transplanted into pots and cultivated under greenhouse conditions for further phenotypic evaluation and seed production.

The sequences of the AtU6 promoter-pegRNA-AtU6 terminator fragment, the CaMV35S-CmYLCV-U6-pegRNA-linker-tevopreQ1-polyT-HSPt fragment, the peanut RPS5A promoter, and the peanut EF1α-1 promoter are provided in Supplementary Texts.

Molecular characterization of the edited lines

Genomic DNA was extracted from peanut calli or leaf tissues using a Super Plant Genomic DNA Kit (Tiangen, Beijing, China). PCR amplification of the *AhALS2* target sites was performed using PrimeSTAR GXL DNA Polymerase (Takara) with primer pairs ALS10F/R and ALS20F/R, respectively, and 100 ng of genomic DNA as the template. The amplification protocol comprised an initial denaturation at 94 °C for 30 s, followed by 30 cycles of 98 °C for 10 s, 55 °C for 15 s, and 68 °C for 3.5 min, with a final extension at 72 °C for 5 min. The PCR products were sent for Sanger sequencing at Sangon Biotech (Shanghai, China). The presence of transgenes in edited T_3 lines was examined by PCR with the following amplification protocol: initial denaturation at 94 °C for 30 s; 30 cycles of 98 °C for 10 s, 55 °C for 15 s, and 68 °C for 1 min (nCas9 fragment), 30 s (hygromycin resistance gene fragment), or 20 s (pegRNA fragment); and a final extension at 72 °C for 5 min.

Primers used for PCR are listed in Table S3.

Herbicide resistance test

The field recommended concentration (FRC) of the commercial herbicides used in this study were as follows: imazethapyr (Shandong Cynda Chemical Corporation Ltd, Weifang, Shandong, China), 0.25 g active ingredient (ai)/L; florasulam (Jiangsu Repont Agrochemical Corporation Ltd, Changzhou, Jiangsu, China), 0.014 g ai/L; bispyribac (Zhenjiang Tianyi Biotechnology Corporation Ltd, Zhengjiang, Jiangsu, China), 0.125 g ai/L; tribenuron-methyl (Anhui Jiatiansen Pesticide Chemical Corporation Ltd, Chizhou, Anhui, China), 0.05 g ai/L; and flucarbazone sodium (Shandong Kangqiao Biotechnology Corporation Ltd, Qingdao, Shandong, China), 0.105 g ai/L. To determine suitable screening concentrations for resistance evaluation, preliminary experiments were conducted to determine the minimum inhibitory concentration (MIC) for each herbicide against WT YH9326 seedlings. Specifically, seedlings were treated with a series of herbicide concentrations at 1/16×, 1/8×, 1/4×, 1/2×, 1×, 2×, 4×, 8×, and 16× FRC. The MIC was defined as the lowest

concentration that caused strong growth inhibition or lethality. The initial screening concentration for tribenuron-methyl was set at 0.005 g ai/L based on a previous study (Shi et al., 2023). The determined MICs were: 0.5 g ai/L for imazethapyr, 0.028 g ai/L for florasulam, 0.125 g ai/L for bispyribac, and 0.105 g ai/L for flucarbazone sodium (Figure S4).

Peanut seeds of both WT and edited lines were sown directly into vermiculite (Zhengzhou NongLe Seedling Matrix Processing Factory) using round plastic pots (9 cm in diameter, 8 cm in height). Plants were cultured in a greenhouse under controlled conditions as described above. One seed was sown per pot, and seedlings were irrigated every other day with tap water. Upon reaching the 3 - 6-leaf stage, peanut seedlings were sprayed once with the designated herbicide at its MIC or a higher concentration, depending on the experimental group. Herbicide application was done using a handheld sprayer to ensure even coverage. Plants were then photographed at 7-day intervals: Day 0 (before treatment), Day 7, Day 14, Day 21, and Day 28 post-treatment. Each treatment was conducted with at least two independent replicates to ensure reproducibility. After 28 days, plant height and root length were measured using a ruler. Seedlings were then placed in paper bags and oven-dried at 37 °C with forced-air circulation until constant weight was achieved for dry biomass determination.

Major agronomic traits evaluation

After 120 days of cultivation in the greenhouse, both mutant and WT YH9326 plants were harvested for agronomic trait assessment. Immediately upon removal from the pots, main shoot length and lateral shoot length were measured using a ruler to evaluate vegetative growth. Following measurement, pods were manually separated, placed in labelled paper bags, and oven-dried with forced air at 37 °C for 3 days to a constant weight. After drying, the kernels were shelled, and the seed yield per plant was determined by weighing the dry seeds. These dried seeds were subsequently used for herbicide resistance assays and oil content analysis.

To evaluate shoot biomass, above-ground parts were excised, transferred to net bags, and dried in a forced-air oven at 37 °C. Shoot dry weight was recorded once a constant weight was reached. Root nodulation traits and fresh weight were assessed within two days after harvest. Specifically, roots were carefully separated from the shoots and stored at 4 °C in sealed plastic bags to preserve moisture. Prior to analysis, root systems were gently rinsed with tap water to remove soil particles, and excess moisture was blotted using filter paper. Nodules were manually collected from each root

system. The total number of nodules and their fresh weight were recorded immediately. Remaining root tissues were transferred to paper bags, oven-dried with forced air at 37 °C, and weighed upon reaching constant mass to determine root biomass. All the above agronomic traits were assessed using a minimum of five biological replicates.

Oil content determination

Harvested peanut seeds were dried in a forced-air oven at 37 °C for three days before being ground into fine powder using a mortar and liquid nitrogen. Three grams of powder were sampled for oil content measurement through the Soxhlet extraction technique (López-Bascón and Luque de Castro, 2020) with diethyl ether as the solvent, following the Chinese Agricultural Industry Standard NY/T 1285-2007: Determination of Oil Content in Oilseeds (Residue Method). Oil content was calculated as the mass of extracted oil divided by the sample weight, multiplied by 100. Each sample was analyzed with three biological replicates and two technical replicates.

Chlorophyll content determination

Leaf chlorophyll content was determined using a handheld SPAD-502Plus chlorophyll meter (Konika Minolta, Inc., Tokyo, Japan) at 32, 50, and 94 days after planting (DAP) (Richardson et al., 2002). For each plant, four measurements per leaflet were taken from the third quadrifoliolate leaves on both the main stem and two lateral branches. SPAD readings were averaged to obtain a representative value per plant. At 32 DAP, most seedlings had only developed the first lateral branch, so measurements at this stage were restricted to quadrifoliolate leaves from the main stem and the first lateral branch. Each mutant line and its corresponding WT control group were assessed using six biological replicates per group.

Off-target effect analysis

Potential off-target sites for the W574 target in *AhALS2* were predicted using the online tool Cas-OFFinder (http://www.rgenome.net/cas-offinder/) (Bae et al., 2014), allowing up to three nucleotide mismatches. Specific primers were designed to amplify these candidate regions (OT1, OT2 and OT3) based on the peanut reference genome (arahy.Tifrunner.gnm2.J5K5) (Table S3). Genomic DNA was extracted from 16 T_3 plants derived from line #150-4-1 and 12 T_3 plants from line #150-3-1 using a Super Plant Genomic DNA Kit (Tiangen). PCR amplification was performed for each candidate off-target site using PrimeSTAR GXL DNA Polymerase (Takara) under the following protocol: initial denaturation at 94 °C for 30 s; 30 cycles of 98 °C for 10 s, 55 °C for 15 s, and 68 °C

for 2 min (OT1), 2.5 min (OT2), or 1 min (OT3); and a final extension at 72 ℃ for 5 min. The resulting products were subjected to Sanger sequencing (Sangon Biotech). The sequencing chromatograms were analyzed to detect any nucleotide substitutions, insertions or deletions at the predicted off-target positions.

Supplementary References

Bae S, Park J, Kim JS (2014) Cas-OFFinder: a fast and versatile algorithm that searches for potential off-target sites of Cas9 RNA-guided endonucleases. Bioinformatics **30:** 1473-1475

Joshi M, Niu C, Fleming G, Hazra S, Chu Y, Nairn CJ, Yang H, Ozias-Akins P (2005) Use of green fluorescent protein as A non-destructive marker for peanut genetic transformation. In Vitro Cellular & Developmental Biology - Plant **41:** 437-445

Lin Q, Jin S, Zong Y, Yu H, Zhu Z, Liu G, Kou L, Wang Y, Qiu JL, Li J, Gao C (2021) High-efficiency prime editing with optimized, paired pegRNAs in plants. Nature Biotechnology **39:** 923-927

López-Bascón MA, Luque de Castro MD (2020) Chapter 11 - Soxhlet Extraction. *In* CF Poole, ed, Liquid-Phase Extraction. Elsevier, pp 327-354

Murashige T, Skoog F (1962) A revised medium for rapid growth and bio assays with tobacco tissue cultures. Physiologia Plantarum **15:** 473-497

Nelson JW, Randolph PB, Shen SP, Everette KA, Chen PJ, Anzalone AV, An M, Newby GA, Chen JC, Hsu A, Liu DR (2022) Engineered pegRNAs improve prime editing efficiency. Nature Biotechnology **40:** 402-410

Richardson AD, Duigan SP, Berlyn GP (2002) An evaluation of noninvasive methods to estimate foliar chlorophyll content. New Phytologist **153:** 185-194

Shi L, Li X, Xue L, Zhang J, Huang B, Sun Z, Zhang Z, Dai X, Han S, Dong W, Zhang X (2023) Creation of herbicide-resistance in allotetraploid peanut using CRISPR/Cas9-meditated cytosine base-editing. Plant Biotechnology Journal **21:** 1923-1925

Wang L, Kaya HB, Zhang N, Rai R, Willmann MR, Carpenter SCD, Read AC, Martin F, Fei Z, Leach JE, Martin GB, Bogdanove AJ (2021) Spelling changes and fluorescent tagging with prime editing vectors for plants. Frontiers in Genome Editing **3**: 617553
